# Biomaterial Engineering for Spatiotemporal Regulation of Exosome Functions: From Design Principles to Key Applications in Regenerative Medicine

**DOI:** 10.3390/ph19050672

**Published:** 2026-04-25

**Authors:** Shan Long, Bo Wang, Shaodong Tian, Honglan Tang, Hanbing Wu, Xiaofeng Yang, Chuyue Zhang

**Affiliations:** 1 Department of Oncology, General Hospital of Hunan University of Medicine, No. 144, Jinxi South Road, Huaihua 418000, China; longshan@hnmu.edu.cn (S.L.);; 2 Department of Nephrology, Institute of Kidney Diseases, West China Hospital, Sichuan University, Chengdu 610041, China; 3 Department of Oncology, The Seventh Medical Center, Chinese PLA General Hospital, No. 5 Nanmencang, Dongcheng District, Beijing 100007, China

**Keywords:** hydrogels, extracellular vesicles, spatiotemporal control, tissue regeneration, smart materials

## Abstract

As natural nanoscale intercellular messengers, exosomes exhibit considerable potential in modulating inflammation, angiogenesis, immunoregulation, and tissue remodeling, making them attractive candidates for regenerative medicine. However, their clinical translation remains limited by rapid systemic clearance, nonspecific biodistribution, insufficient lesion retention, and functional attenuation in hostile pathological microenvironments. In this review, we propose that biomaterial engineering should evolve from providing passive exosome carriers to constructing active regulatory platforms capable of precise spatiotemporal control. We summarize engineering strategies along two complementary dimensions. In the temporal dimension, biomaterials can enable sustained, sequential, or microenvironment-responsive release to match the dynamic phases of tissue repair. In the spatial dimension, biomaterials can improve local retention, tissue anchoring, structural guidance, endogenous cell recruitment, and lesion-specific delivery. Using cutaneous wound healing, osteochondral regeneration, myocardial repair, and neural regeneration as representative examples, we further analyze these strategies through a “clinical challenge–engineering strategy–biological mechanism” framework, with particular attention to how engineered systems influence key signaling pathways such as PI3K/Akt, Wnt/β-catenin, NF-κB, and PTEN/PI3K/Akt/mTOR. We also discuss translational barriers, including exosome heterogeneity, safety concerns inherited from parental cells, large-scale GMP-compliant manufacturing, product standardization, storage stability, and regulatory classification of exosome–biomaterial hybrids. Finally, we highlight emerging directions, including multi-mechanism combinational systems, closed-loop responsive platforms, and artificial intelligence-assisted design for personalized exosome therapeutics. This review provides a design-oriented framework to accelerate the bench-to-bedside development of biomaterial-enabled precision exosome therapy.

## 1. Introduction: The Engineering Challenge of Exosome Therapy—From “Panacea” to “Precision Missile”

### 1.1. The Potential of Exosomes and Their Delivery Dilemma

The ultimate goal of regenerative medicine is to repair, replace, or regenerate human tissues and organs. In this context, exosomes, as the “messengers” through which stem cells exert their paracrine functions, are spearheading a paradigm shift towards “cell-free therapy” [1,2,3,4]. These extracellular vesicles, with diameters ranging from 30 to 150 nm, deliver their rich cargo of bioactive molecules—such as proteins, mRNAs, and miRNAs—to target cells [5,6,7,8]. This delivery allows them to play a multidimensional regulatory role in tissue repair, including potent anti-inflammatory, immunomodulatory [9], pro-angiogenic [10], and pro-proliferative/differentiative functions [11]. Crucially, they circumvent the inherent risks associated with direct cell transplantation, such as immune rejection and tumorigenicity [12]. Crucially, exosomes avoid several risks associated with viable cell transplantation, such as uncontrolled engraftment or ectopic proliferation. However, they should not be regarded as biologically inert products, because their membrane composition and molecular cargo can partially reflect the state of the parental cells and may therefore induce unintended immunomodulatory or even tumor-promoting effects in certain contexts [13,14,15].

Compared with conventional drug delivery systems, exosome-based platforms offer several distinctive advantages, including intrinsic membrane biocompatibility, the ability to carry complex biological cargo, partial capacity to cross biological barriers, and endogenous intercellular communication properties [13]. Nevertheless, these advantages are accompanied by important limitations, such as source-dependent heterogeneity, limited manufacturing standardization, and unclear potency criteria. Therefore, the key value of combining exosomes with biomaterials is not merely to prolong residence time, but to integrate the biological sophistication of exosomes with the structural, mechanical, and programmable features of engineered materials. From this perspective, biomaterial–exosome systems should be regarded as hybrid therapeutic platforms rather than simple sustained-release formulations [16].

However, the therapeutic efficacy of directly and systemically administering exosomes in clinical settings has fallen far short of expectations [17]. This gap between theory and reality stems from the inherent delivery challenges of exosomes: (1) Extremely short half-life: Following intravenous injection, exosomes are rapidly cleared by the mononuclear phagocyte system [18]. (2) Insufficient targeting: Exosomes lack the ability to actively target pathological sites, leading to excessively low local effective concentrations [19,20]. (3) Microenvironment-induced inactivation: The bioactivity of exosomes is easily compromised in complex pathological microenvironments, such as those with high oxidative stress or acidic conditions [10]. A core scientific challenge in this field is how to engineer these natural “nanomessengers” into “biological missiles” that can precisely strike diseased tissues and exert their effects efficiently within a predetermined time and space.

A clearer understanding of exosome pharmacokinetics further explains why biomaterial assistance is needed. Following systemic administration, exosomes are often rapidly sequestered by the mononuclear phagocyte system, with preferential accumulation in the liver and spleen [18,21]. Their in vivo biodistribution is strongly influenced by donor cell source, vesicle surface composition, route of administration, and targeting modification [22]. As a result, only a limited fraction of administered exosomes reaches the lesion site, especially in deeply located or poorly vascularized tissues. These pharmacokinetic constraints provide a strong rationale for localized biomaterial-assisted delivery, which can improve lesion retention, reduce off-target exposure, and reshape the local therapeutic concentration–time profile [23].

### 1.2. The Evolving Role of Biomaterials: From Passive Carriers to Active Modulators

The intervention of biomaterial engineering offers elegant and powerful solutions to these challenges. Initially, biomaterials were introduced into exosome delivery systems as Passive Carriers. By physically encapsulating or chemically conjugating exosomes within hydrogels, scaffolds, and other materials, researchers achieved in situ retention and sustained release [24,25]. This approach directly addresses the important issues of short half-life and low target concentration by maintaining a therapeutically effective dose at the local site of injury [26,27].

Nevertheless, a superior biomaterial design should function as an Active Modulator, not only delivering exosomes but also engaging in dynamic and intelligent interactions with them, host cells, and the pathological microenvironment. This evolution in its role is manifested on two levels. First, through sophisticated chemical design, materials can sense specific signals in the pathological microenvironment (e.g., pH, reactive oxygen species (ROS), matrix metalloproteinases (MMPs)) to achieve “on-demand” release of exosomes [28,29]. Second, advanced manufacturing techniques, such as 3D printing, can endow materials with specific biomimetic architectures that provide physical guidance for orderly cell regeneration [30,31]. In this “active modulation” paradigm, the biomaterial is no longer a mere vehicle but an indispensable functional module within the therapeutic system, working synergistically with exosomes to maximize therapeutic outcomes.

Accordingly, the central question in this field is no longer whether biomaterials can carry exosomes, but how biomaterials can be rationally designed to regulate when, where, and under which pathological conditions exosomes should act. This shift from “delivery” to “programmed regulation” also provides the conceptual basis for distinguishing the present review from earlier summaries focused mainly on material categories or isolated application scenarios.

### 1.3. Core Thesis and Structure of This Review

Building on this framework, the present review aims to move beyond the conventional view of biomaterials as passive exosome depots. Unlike recent reviews that primarily summarize exosome engineering, hydrogel-based delivery, or application-specific studies in bone, skin, or nerve repair [13,16,24,32], this article uses spatiotemporal regulation as the central organizing principle. Specifically, we propose that biomaterial design should be interpreted through two interconnected dimensions: temporal programming, which determines release rhythm and therapeutic sequence, and spatial programming, which governs retention, localization, tissue guidance, and lesion responsiveness. On this basis, we construct a design-oriented framework linking engineering principles, cargo-mediated biological mechanisms, major regenerative applications, and clinical translation barriers. Using cutaneous, osteochondral, myocardial, and neural repair as representative settings, we discuss how distinct biomaterial strategies can be adapted to different pathological contexts and therapeutic objectives.

## 2. The Engineering “Toolbox” for Spatiotemporal Control of Exosome Function

To shift exosome application from a “shotgun” approach to a “precision strike” strategy, researchers have developed a series of sophisticated biomaterial designs, forming a powerful “engineering toolbox.” These strategies can be categorized along two complementary dimensions—Temporal and Spatial—which work in concert to achieve unprecedentedly precise control over exosome bio-functions.

**Figure 1:**
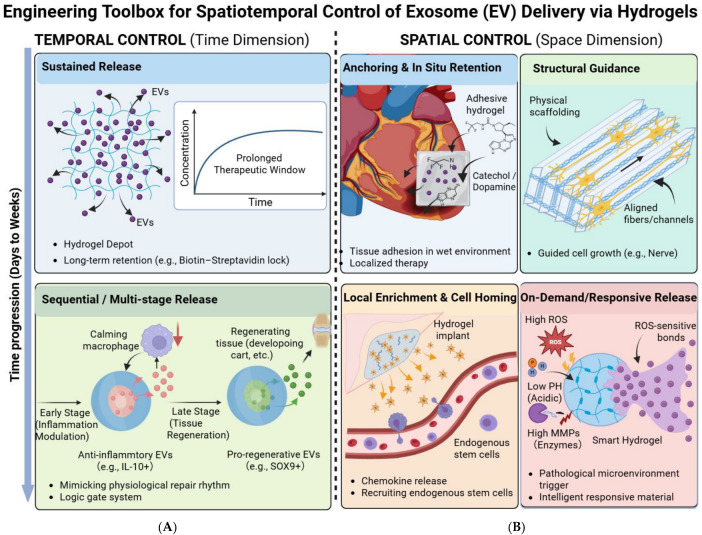
Schematic illustration of the engineering toolbox for spatiotemporal control of exosome (EV) delivery via hydrogels. The diagram summarizes strategies for precise EV delivery across two dimensions: (**A**) Temporal Control (Left panel): Strategies include “Sustained Release” to prolong the therapeutic window (e.g., via hydrogel depots or affinity locking) and “Sequential/Multi-stage Release” to mimic physiological repair rhythms (transitioning from inflammation modulation to tissue regeneration). (**B**) Spatial Control (Right panel): Strategies encompass “Anchoring & In Situ Retention” using adhesive hydrogels, “Structural Guidance” provided by physical scaffolding (e.g., aligned fibers), “Local Enrichment” via endogenous stem cell homing, and “On-Demand/Responsive Release” triggered by pathological microenvironmental cues (e.g., high ROS, acidic pH, or enzymes). For translational relevance, future versions of such platforms should also be evaluated using quantitative parameters such as loading efficiency, burst release ratio, retention half-life, and lesion-site fluorescence persistence.

Importantly, these strategies are not mutually exclusive. In practice, clinically useful systems often combine temporal and spatial programming to match the biological and physical constraints of a given indication. For example, chronic wounds require inflammatory microenvironment-responsive release and irregular-shape filling; osteochondral defects require structural guidance and mechanical support; myocardial repair requires minimally invasive delivery with strong local retention under dynamic motion; and neural repair requires directional guidance together with long-term neurotrophic support. Therefore, the engineering toolbox should be understood as a modular framework that can be tailored to specific therapeutic scenarios rather than a collection of isolated material techniques (Figure 1).

### 2.1. Temporal Control

The essence of temporal control lies in managing the “rhythm” and “timing” of exosome release at the site of injury.

#### 2.1.1. Sustained Release

This is the most fundamental and widely used temporal control strategy. Its objective is to create an “exosome depot” at the injury site to maintain a therapeutic concentration for days or even weeks. Injectable hydrogels are ideal carriers for this purpose, including thermo-responsive chitosan/β-glycerophosphate (CS/GP) [33] or Pluronic F-127 [27] hydrogels, as well as photocurable gelatin methacryloyl (GelMA) [34] or extracellular matrix (ECM)-based hydrogels [26]. To achieve even stronger retention and longer release profiles, strategies based on high-affinity biorecognition have been developed. For instance, the biotin–streptavidin system has been used to firmly “lock” exosomes within a hydrogel network, achieving sustained retention and release for up to 28 days [35].

Beyond conceptual sustained release, the quantitative improvement in retention kinetics is especially important for translational evaluation. In many hydrogel systems, passive physical encapsulation reduces early burst loss and extends detectable exosome retention from hours to several days, whereas affinity-mediated strategies can further prolong local retention to weeks. For example, high-affinity biorecognition systems such as biotin–avidin interactions markedly suppress initial diffusion-driven loss and maintain higher local exosome exposure over extended periods [35]. However, stronger retention is not always synonymous with better efficacy. Excessively tight binding may delay early bioavailability, and the optimal balance depends on the pathological stage, local fluid dynamics, and the intended therapeutic window [28]. Therefore, affinity engineering should be viewed as a tunable parameter rather than a universally superior design.

#### 2.1.2. Sequential/Multi-Stage Release

Complex tissue repair is a highly ordered, multi-stage biological event [36,37]. A more advanced temporal control strategy involves mimicking this natural reparative rhythm by delivering therapeutic factors with different functions in a chronological sequence. A highly representative study developed a “logic gate” system composed of an MMP-13-sensitive peptide-crosslinked hydrogel and microspheres encapsulating PGE_2_. This system achieved the preferential release of anti-inflammatory IL-10^+^ EVs in the early phase of cartilage repair, followed by the later release of pro-chondrogenic SOX9^+^ EVs, perfectly replicating the physiological sequence of repair [34]. Similarly, microneedle arrays with a core–shell structure can achieve sequential delivery by first rapidly releasing a surface-loaded anti-inflammatory small molecule, followed by the sustained release of exosomes from the core [38].

Sequential release systems are particularly valuable because tissue repair is not a static process [37]. Early-stage control of excessive inflammation, mid-stage promotion of angiogenesis or progenitor activation, and late-stage support of matrix remodeling or lineage-specific differentiation often require different signals at different times [38]. Accordingly, the design goal is not simply prolonged release, but temporally differentiated bioavailability. Recent systems have demonstrated that dual-factor or dual-EV release can be programmed through differential crosslink degradation, layered architectures, or core–shell devices [34]. Such designs better approximate physiological healing rhythms and may reduce pathway interference that can occur when anti-inflammatory and pro-regenerative signals are delivered simultaneously at non-optimized ratios.

### 2.2. Spatial Control

The core of spatial control is to manage the “position,” “distribution,” and “target” of exosomes in three-dimensional space. At the molecular level, spatial control depends not only on macroscopic scaffold placement but also on physicochemical interactions between exosomes and biomaterial networks. These interactions may include electrostatic adsorption, hydrophobic association, hydrogen bonding, catechol-mediated adhesion, aptamer-based recognition, and high-affinity ligand–receptor pairing. In addition, network pore size, swelling ratio, and crosslink density directly influence exosome diffusivity and release behavior [21,32,39]. Therefore, spatial regulation is fundamentally a multiscale phenomenon, spanning nanoscale binding events, microscale transport, and macroscale tissue localization.

#### 2.2.1. Anchoring & In Situ Retention

For tissues in dynamic or wet environments, such as the myocardium or cartilage, ensuring that the delivery system can firmly “adhere” to the lesion surface is critical. Inspired by mussel adhesive proteins, researchers have incorporated catechol-rich chemical structures (like dopamine) into hydrogels to develop materials with excellent tissue adhesiveness. These hydrogels can bind tightly to tissue surfaces, achieving stable in situ anchoring [32,40]. For example, an adhesive hyaluronic acid hydrogel was successfully applied to the wall of the infarct cavity after a stroke, effectively achieving local retention and functional deployment of exosomes [41].

Nevertheless, improved adhesion and in situ retention also introduce design trade-offs. Strong tissue adhesion can enhance lesion coverage and reduce washout in wet or mechanically active environments, but overly dense adhesive interactions may impede exosome diffusion into surrounding tissue. Similarly, patch-like retention is advantageous for myocardium, cartilage, or stroke cavities, yet may be less suitable when deep tissue penetration or broad interface coverage is required. Therefore, anchoring strategies should be optimized according to tissue mobility, fluid exposure, and desired penetration depth.

#### 2.2.2. Structural Guidance & Cell Organization

Biological tissues possess highly precise three-dimensional structures. By endowing biomaterials with specific physical topographies using advanced manufacturing techniques, a “physical scaffold” can be provided for cell regeneration [42,43]. In the field of nerve regeneration, conductive hydrogels with oriented grooves or fibers can guide neurite extension along a predefined path [31,44]. In bone-cartilage repair, 3D printing can create bilayer scaffolds with gradient porosity and composition, guiding the spatially specific differentiation of stem cells [30,45]. One study even combined a 3D-printed biomimetic trabecular titanium alloy scaffold with an exosome-laden hydrogel, unifying mechanical support and biological activity [46].

#### 2.2.3. Local Enrichment & Cell Homing

A spatial control strategy involves designing biomaterials that not only deliver exosomes but also actively “recruit” endogenous stem cells to the injury site [47]. By integrating chemoattractants or specific recognition peptides into the material, circulating or surrounding stem cells can be attracted to migrate and accumulate at the lesion [32]. One study covalently crosslinked a stem cell-specific binding peptide onto hydrogel microspheres, achieving a synergistic effect of efficient endogenous stem cell capture and exosome delivery, demonstrating the therapeutic potential of this dual-action approach [48].

#### 2.2.4. On-Demand Release in Response to the Pathological Microenvironment

This is the most “intelligent” strategy in spatiotemporal control. Its core principle is to enable the material to “read” characteristic signals of the pathological microenvironment and release exosomes “on-demand.” By using signals unique to the pathological state (e.g., low pH, high ROS, high MMPs) as triggers, smart responsive materials can be designed [49,50,51,52]. For instance, by incorporating ROS-sensitive chemical bonds (such as boronate esters), the material degrades and releases exosomes when exposed to the high ROS environment of a chronic wound, providing the most timely treatment when inflammation is most severe [29,53]. Similarly, acid-labile chemical bonds can be used to achieve pH-responsive “on-demand release” [28].

From a materials perspective, responsive release is governed by stimulus-coupled network destabilization. In ROS-responsive systems, boronate ester or thioketal-containing linkers are cleaved under oxidative stress, thereby accelerating matrix degradation and exosome liberation [29]. In acidic environments, protonation-sensitive or acid-labile bonds can weaken network integrity and trigger pH-dependent release [28]. Enzyme-responsive systems, particularly those sensitive to MMP-rich pathological niches, exploit cleavage of peptide crosslinks to achieve lesion-specific degradation. Importantly, these degradation mechanisms not only determine release kinetics but also influence local mechanics, diffusion pathways, and the temporal coupling between microenvironment normalization and exosome exposure.

### 2.3. Engineering Exosomes Themselves for Spatial and Temporal Therapeutic Targeting

#### 2.3.1. Programming Biodistribution and Organ Tropism

Biomaterials define where and when exosomes are released, whereas exosome-level engineering influences where they distribute after administration and which organs or tissues they preferentially reach. In vivo biodistribution is strongly affected by donor-cell source, route of administration, and dose. After systemic delivery, a substantial fraction of extracellular vesicles is commonly sequestered by the liver, spleen, and lungs, which limits delivery to diseased tissues [22,23]. Accordingly, the route of administration should be matched to the anatomical accessibility of the lesion: local injection or topical implantation is generally preferable for focal regenerative lesions, whereas systemic administration may be required for deep or disseminated targets. When intravenous delivery is unavoidable, engineering strategies that reduce mononuclear phagocyte system (MPS) uptake or extend circulation time become particularly important. For example, transient MPS blockade significantly improved myocardial delivery and therapeutic efficacy of exosomes in vivo [18]. In parallel, surface display of targeting ligands provides a more direct approach to organ or cell-specific delivery, as illustrated by RVG-modified exosomes for brain delivery and EGFR-targeted exosomes for receptor-enriched cells [54,55].

#### 2.3.2. Enhancing Cell-Specific Uptake and Local Retention

Once exosomes reach the lesion site, their therapeutic efficacy further depends on efficient uptake by the intended recipient cells. This step can be enhanced by membrane engineering through genetic modification of donor cells; post-isolation chemical conjugation; glycoengineering; or the display of peptides, aptamers, and nanobodies that promote receptor-mediated internalization [56,57,58]. Surface engineering may also be combined with pharmacokinetic optimization. PEGylated and targeted extracellular vesicles, for instance, have shown enhanced cell specificity together with prolonged circulation time, highlighting the feasibility of simultaneously tuning biodistribution and uptake efficiency [56]. For local regenerative therapies, retention can be further reinforced by affinity-mediated immobilization within biomaterials. A representative example is the biotin–avidin system, which enabled sustained release for 7 days in vitro and retention for up to 28 days in vivo, thereby increasing local exosome exposure and improving tissue repair outcomes [35]. Likewise, targeting peptide-modified engineered exosomes embedded in photocrosslinked hydrogels improved therapeutic performance in osteoarthritis, illustrating how exosome engineering and biomaterial design can act synergistically [19].

#### 2.3.3. Temporal Programming According to Disease Stage

Temporal precision depends not only on release kinetics but also on matching exosome function to the dynamic biology of tissue repair. Parent-cell preconditioning and cargo engineering are therefore important strategies for programming stage-specific activity. Hypoxic preconditioning can enrich exosomes with pro-angiogenic and cytoprotective signals, making them suitable for ischemic or poorly vascularized wounds [10]. Mechanical preconditioning of BMSCs with the Piezo1 agonist Yoda1 enhances the osteogenic activity of their exosomes, which is particularly relevant for bone regeneration [59]. In spinal cord injury, 3D culture and dexamethasone loading were used to reinforce the anti-inflammatory, neurotrophic, and pro-angiogenic effects of EVs before their incorporation into an ROS-responsive hydrogel, thereby coupling exosome optimization with microenvironment-triggered release [29]. A more advanced example is the logic-gated hydrogel system that sequentially releases IL-10^+^ EVs during the early inflammatory phase and SOX9^+^ EVs during the later reparative phase, closely recapitulating the biological sequence from immunomodulation to tissue reconstruction [34]. These studies indicate that temporal engineering should be understood as a combination of vesicle programming and release programming.

#### 2.3.4. Adapting Engineering Strategies to Different Clinical Scenarios

Importantly, no single engineering strategy is universally optimal. Instead, the design of exosomes should be adapted to lesion accessibility, dominant biological barriers, disease stage, and the intended therapeutic window. For superficial and irregular wounds, injectable hydrogels combined with pro-angiogenic or immunomodulatory exosomes are advantageous because they improve local retention while allowing responsive release in acidic or ROS-rich microenvironments [10,28]. For osteochondral defects, strong local fixation, wet-tissue adhesion, and osteo/chondrogenic programming are more important because these lesions are mechanically loaded and spatially heterogeneous [19,32]. For myocardial repair, local catheter-based or intramyocardial delivery is often preferable, whereas systemic administration may additionally require MPS avoidance and active heart-targeting strategies to overcome rapid hepatic and splenic sequestration [18]. For neural indications, the engineering priority depends on the delivery route: BBB-penetrating ligands are valuable for systemic brain delivery, while locally implanted aligned or conductive biomaterials are more suitable for focal spinal cord injury [29,31,54]. Collectively, these findings support a scenario-adapted framework in which exosome engineering is selected according to the spatial and temporal therapeutic target rather than applied as a uniform platform.

## 3. Application and Mechanistic Analysis Guided by Design Principles

The ultimate value of the “engineering toolbox” lies in solving specific clinical problems. This section will use four major applications in regenerative medicine as examples to dissect how the aforementioned spatiotemporal design principles are applied to formulate advanced therapeutic strategies.

To avoid interpreting signaling pathways as black-box outcomes, it is important to distinguish three mechanistic layers in these systems: (i) biomaterials regulate exosome retention, release, and cellular uptake; (ii) exosomal cargo, including specific miRNAs, proteins, or lipids, interacts with defined molecular nodes in recipient cells; and (iii) these intracellular events converge on pathway-level changes that shape inflammation resolution, angiogenesis, matrix remodeling, and lineage differentiation. In this sense, biomaterials do not merely deliver exosomes; they reshape the timing, localization, and magnitude of cargo-mediated signaling.

**Figure 2:**
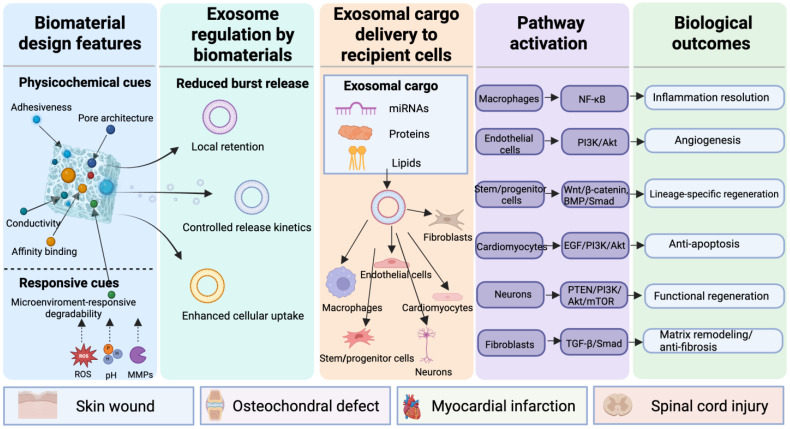
Mechanistic framework linking biomaterial design, exosome delivery behavior, cargo-mediated signaling, and regenerative outcomes. Biomaterial engineering regulates exosome therapy through two interconnected dimensions: temporal programming and spatial programming. Temporal strategies, including sustained, sequential, and microenvironment-responsive release, determine when exosomes are available at the lesion site. Spatial strategies, including tissue anchoring, in situ retention, structural guidance, local enrichment, and conductive/aligned support, determine where exosomes accumulate and how effectively they interact with target cells. These material-enabled controls reshape exosome fate by improving local retention, reducing burst loss, preserving bioactivity, and enhancing cellular uptake. Delivered exosomal cargo, such as miRNAs, proteins, lipids, and other bioactive molecules, acts on recipient cells including macrophages, endothelial cells, fibroblasts, stem/progenitor cells, cardiomyocytes, and neurons/glial cells. These interactions modulate key signaling pathways, including NF-κB, PI3K/Akt, Wnt/β-catenin, BMP/Smad, EGF/PI3K/AKT, PTEN/PI3K/AKT/mTOR, and TGF-β/Smad, thereby promoting inflammation resolution, angiogenesis, anti-apoptosis, anti-fibrosis, matrix remodeling, and functional tissue regeneration across cutaneous, osteochondral, myocardial, and neural repair settings.

To improve readability and cross-study comparison, Table 1 summarizes representative biomaterial-enabled exosome delivery systems according to biomaterial type, exosome source, engineering strategy, release/retention behavior, model system, therapeutic effect, and major translational limitation (Figure 2).

### 3.1. Skin Wound Healing (Especially Chronic Wounds)

Clinical Challenge: The healing of chronic wounds, such as diabetic foot ulcers, is a formidable clinical problem [63]. Its core pathology is a vicious cycle of persistent inflammation, severe angiogenic impairment, and cellular dysfunction [64,65]. To break this self-sustaining pathological stalemate, engineering strategies must be able to intelligently intervene in the wound microenvironment and adjust the therapeutic rhythm according to its dynamic changes. Accordingly, for superficial but highly heterogeneous wounds, the preferred scenario-adapted strategy is to combine locally injectable, ROS/pH-responsive hydrogels with exosomes selected or engineered for immunomodulatory and pro-angiogenic activity, thereby matching exosome presentation to the inflammatory and reparative phases of healing [10,28,57].

Engineering Strategy and Application: For irregular wound shapes and complex pathological microenvironments, injectable, smart responsive hydrogels (applying Section 2.2.1 and Section 2.2.4) are the mainstream strategy. These materials not only fill the wound but also “sense” the microenvironment and release drugs “on-demand.” For example, Wang et al. developed a pH-responsive self-healing hydrogel that accelerates its degradation to release MSC-derived exosomes in the characteristic acidic environment of diabetic wounds [28]. These exosomes, rich in anti-inflammatory miRNAs, promote the polarization of macrophages from the M1 to M2 phenotype by inhibiting the NF-κB signaling pathway, thus breaking the inflammatory deadlock [35,66]. Following inflammation resolution, the sustained release of pro-angiogenic factors (e.g., VEGF) and miRNAs (e.g., miR-126) from the exosomes promotes neovascularization by activating the PI3K/Akt pathway in endothelial cells, improving wound ischemia [10,67]. More recently, sophisticated ROS-responsive hydrogels have been developed that use high oxidative stress as a trigger for exosome release, achieving precise temporal control by administering the therapeutic when inflammation is most intense [29,53]. Other researchers have designed multifunctional hydrogels loaded with MnO_2_ nanosheets, which not only responsively release exosomes but also catalyze the conversion of excess H_2_O_2_ in the wound into oxygen, achieving the dual functions of “smart release” and “hypoxia alleviation” to synergistically accelerate the healing process [60].

Mechanistically, these wound-repair systems act through a coordinated sequence of immunomodulation, angiogenesis, and tissue remodeling [10]. Exosomal cargo such as anti-inflammatory miRNAs can suppress NF-κB-associated macrophage activation and promote M2 polarization, whereas pro-angiogenic cargo enhances endothelial migration, tube formation, and vascular maturation through PI3K/Akt-related signaling. Importantly, biomaterials influence not only how much exosome reaches the wound, but also when these signals dominate during healing [28]. From a translational perspective, exosome-based interventions for wound healing have already entered early clinical evaluation, although most reported studies involve EVs alone or relatively simple formulations rather than fully integrated exosome–hydrogel combination products [68]. This gap highlights both the promise and the current developmental immaturity of biomaterial-assisted exosome therapy in cutaneous regeneration [69].

### 3.2. Bone/Cartilage Regeneration

Clinical Challenge: The repair of large-segment bone defects or articular cartilage damage is extremely challenging because these tissues have poor self-repair capacity and must withstand complex mechanical loads [70]. Successful treatment requires not only long-term osteogenic/chondrogenic inductive signals but also a structurally and mechanically suitable “scaffold” [71]. Therefore, an ideal engineering strategy must satisfy the dual needs of “biological signaling” and “physical support.” Advanced biomanufacturing provides a perfect solution. Accordingly, osteochondral repair requires a scenario-adapted design that integrates mechanically competent and spatially organized scaffolds with exosomes engineered for osteogenic/chondrogenic induction and prolonged local retention under load-bearing conditions [19,30,59,72].

Engineering Strategy and Application: The mainstream strategy to address this challenge is the use of 3D-printed porous scaffolds (applying Section 2.2.2) that facilitate the long-term sustained release of exosomes (applying Section 2.1.1). A landmark study utilized 3D printing to construct a bilayer gradient hydrogel scaffold based on decellularized extracellular matrix (dECM). Its porous structure facilitates cell ingrowth, while the layered design mimics cartilage and bone tissues. The sustained release of loaded exosomes guided spatially specific tissue regeneration by activating osteogenic pathways like BMP/Smad or Wnt/β-catenin in stem cells, successfully repairing full-thickness osteochondral defects in rats [30,58]. To address the load-bearing issue, some studies have combined 3D-printed biomimetic trabecular titanium alloy scaffolds with exosome-loaded hydrogel microspheres, achieving a powerful combination of excellent mechanical properties and bioactivity [46,73]. In addition to optimizing the material, optimizing the exosomes themselves is an important direction. Genetically engineering parent cells to overexpress Bmp2 can produce engineered exosomes with enhanced osteogenic activity [72]. A recent innovative study found that pre-conditioning BMSCs with mechanical stimulation (using the Piezo1 agonist Yoda1) significantly enhanced the osteogenic activity of their secreted exosomes, offering a new approach to improving therapeutic efficacy from the source [59]. These exosomes can also effectively modulate the bone immune microenvironment, creating favorable conditions for osteogenesis [74].

For osteochondral and bone applications, mechanical performance is not a secondary consideration but a core design determinant. Scaffolds intended for osteochondral repair must provide sufficient compressive resistance, interfacial stability, and pore interconnectivity while still permitting nutrient transport and exosome diffusion [30]. In the current literature, reported mechanical indices vary widely because of differences in material composition, hydration state, testing geometry, and loading protocol, which complicates direct cross-study comparison [24,46]. Nevertheless, available evidence consistently indicates that the most successful systems are those that balance biological activity with architecture-dependent mechanical support. This is particularly important in layered or gradient constructs, where each region should more closely approximate the functional demands of cartilage versus subchondral bone [45].

### 3.3. Myocardial Repair

Clinical Challenge: Following myocardial infarction (MI), myocardial tissue is non-regenerative. The pathological process primarily involves massive apoptosis of cardiomyocytes in the infarct zone, scar formation due to fibrosis, and destruction of the local vascular network [75]. Addressing the trifecta of apoptosis, fibrosis, and vascular damage within the continuously beating and hard-to-reach heart poses extremely high demands on the delivery system’s minimal invasiveness, targeting ability, and multifunctionality. Accordingly, because myocardial lesions are deep, dynamic, and difficult to access, the preferred strategy is local catheter-based or intramyocardial delivery with in situ-forming adhesive depots, whereas systemic administration, if required, should be combined with circulation optimization and active targeting to reduce mononuclear phagocyte system sequestration and off-target loss [18,39,56,61].

Engineering Strategy and Application: Given these challenges, injectable, tissue-adhesive, in situ-forming hydrogels (applying Section 2.1.1 and Section 2.2.1) represent the most ideal delivery platform. Delivered via catheter-based injection to the infarct area, the material rapidly gels to form a therapeutic “patch.” A study published in PNAS utilized a DNA hydrogel for the sustained release of MSC exosomes. These exosomes improved cardiac function in MI rats by upregulating the Bcl-2/Bax ratio to inhibit cardiomyocyte apoptosis and suppressing the TGF-β1/Smad2/3 pathway to reduce fibrosis [39]. Particularly noteworthy is a synergistic therapeutic strategy combining “biochemical” and “physical” signals. This study developed a conductive polypyrrole/chitosan (PPY-CHI) hydrogel. It not only exerts anti-apoptotic and pro-angiogenic effects via sustained exosome release (activating the EGF/PI3K/AKT pathway) but also conducts myocardial electrical signals to synchronize ventricular contraction and ameliorate arrhythmia, offering a new multimodal therapeutic perspective for myocardial repair [61].

For cardiac applications, biomaterial-assisted localization is especially important because freely injected exosomes are rapidly dispersed or cleared in the highly perfused and continuously contracting myocardium [18]. Tissue-adhesive and injectable hydrogels therefore serve two functions simultaneously: they improve local pharmacokinetics and create a permissive niche for sustained paracrine signaling. However, myocardial repair also illustrates a broader translational challenge in the field: multifunctional designs that combine conductivity, adhesiveness, injectability, and exosome release may yield superior biological outcomes, but they also increase manufacturing complexity, quality-control burden, and regulatory uncertainty [39,61,76].

### 3.4. Nerve Regeneration

Clinical Challenge: The repair of nerve injury, especially spinal cord injury (SCI), is the “holy grail” of regenerative medicine [77]. The main obstacles include neuronal death and axonal transection, physical and chemical inhibition of axonal regeneration by the glial scar, and the lack of proper directional guidance for nascent axons [78,79]. Therefore, an effective strategy must be a “triune” comprehensive solution that simultaneously provides neurotrophic support, overcomes the inhibitory microenvironment, and offers physical guidance. Accordingly, neural indications require the most stringent spatial design: focal spinal cord injury is better served by locally implanted aligned or conductive biomaterials with sustained exosome presentation, whereas brain-directed therapies may additionally require exosome surface-targeting strategies to cross the blood–brain barrier after systemic administration [31,54,62].

Engineering Strategy and Application: Based on this concept, the mainstream strategy involves constructing conductive scaffolds/hydrogels with oriented micro/nanostructures (applying Section 2.2.2) and achieving long-term sustained release of exosomes (applying Section 2.1.1). A classic study in Advanced Materials used a conductive hydrogel loaded with BMSC exosomes to repair SCI. Its oriented structure provided physical guidance for axonal regeneration, while its conductivity promoted electrical signal transmission. Concurrently, the sustained release of exosomes chemically promoted effective axonal regeneration by providing neurotrophic factors and activating neuronal growth pathways such as PTEN/PI3K/AKT/mTOR [31]. For more intelligent therapy, another study designed a ROS-responsive hydrogel that releases engineered exosomes “on-demand” within the intense inflammatory microenvironment post-SCI [29]. Furthermore, precision strategies are emerging. One study used single-cell sequencing to screen for the CD271^+^CD56^+^ subpopulation of BMSCs with the strongest pro-regenerative capacity and discovered that their exosomes act via a unique miR-431-3p/RGMA axis. This represents a precision medicine approach of optimizing exosome function through parent cell screening [62]. Meanwhile, the sources of exosomes are broadening; for example, research has explored the use of plant-derived ginseng exosomes in promoting neural stem cell differentiation, opening new frontiers for the field [80].

**Figure 3:**
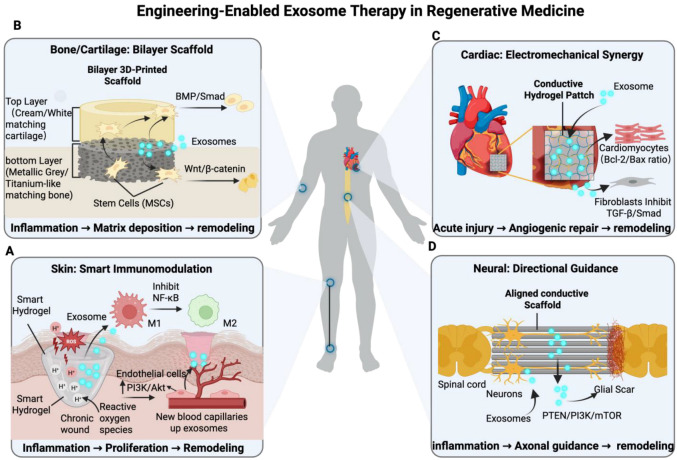
Schematic representation of engineering-allowed exosome therapy across major regenerative medicine applications. The diagram illustrates how engineered hydrogel systems are tailored to specific tissue pathologies to achieve precise exosome delivery and therapeutic outcomes. (**A**) Skin Regeneration: Smart Immunomodulation. In chronic wounds, “smart” hydrogels respond to pathological triggers such as high reactive oxygen species (ROS) and acidic pH (H+) to release exosomes. These exosomes promote the transition of macrophages from the pro-inflammatory M1 to the anti-inflammatory M2 phenotype via NF-κB inhibition and stimulate endothelial angiogenesis through the PI3K/Akt pathway. (**B**) Bone/Cartilage Regeneration: Bilayer Scaffolds. A 3D-printed bilayer scaffold mimics the native osteochondral interface, with distinct layers matching cartilage (top) and bone (bottom). The scaffold directs the differentiation of mesenchymal stem cells (MSCs) via the BMP/Smad and Wnt/β-catenin pathways, respectively, facilitating simultaneous osteochondral repair. (**C**) Cardiac Repair: Electromechanical Synergy. Conductive hydrogel patches restore electrical coupling in infarcted hearts while delivering exosomes to modulate cellular functions. The therapy inhibits cardiomyocyte apoptosis by increasing the Bcl-2/Bax ratio and reduces fibrosis by suppressing TGF-β/Smad signaling in fibroblasts. (**D**) Neural Regeneration: Directional Guidance. For spinal cord injury, aligned conductive scaffolds provide physical guidance to bridge the gap across the glial scar. Synergistically, delivered exosomes activate the PTEN/PI3K/mTOR pathway to promote directional axonal regrowth and functional reconnection. Biological timelines indicating the dominant repair phases (inflammation resolution, angiogenesis/progenitor activation, and remodeling) should be incorporated to better visualize the temporal rationale of each engineering strategy.

Notably, the therapeutic logic in neural repair is particularly dependent on spatiotemporal coordination. Early modulation of the inflammatory milieu and oxidative stress may preserve vulnerable neurons and reduce secondary injury, whereas later directional guidance and neurotrophic support are required for axonal extension and reconnection [62]. In this context, conductive or aligned biomaterials amplify the action of exosomal cargo by increasing the probability that biochemical signaling and physical guidance occur in the same regenerative corridor [29,31]. This may partly explain why exosome-loaded oriented conductive hydrogels outperform non-structured delivery systems in spinal cord injury models (Figure 3).

## 4. Conclusions, Challenges, and Future Perspectives

### 4.1. Summary

This review has systematically described how biomaterial engineering, through sophisticated spatiotemporal design, may help develop exosome-based therapeutics with more precise delivery precisely addressing specific clinical challenges. We have constructed an “engineering toolbox” and, through application examples in four major regenerative medicine fields, demonstrated how these design principles combine with profound biological mechanisms to form a complete logical loop: Clinical Problem → Engineering Design → Core Mechanism → Functional Regeneration.

### 4.2. Challenges in Clinical Translation

Despite substantial progress in laboratory research, translating exosome-based therapeutics into clinically viable products still faces a substantial “valley of death”. This challenge is particularly pronounced for biomaterial-integrated exosome systems, in which both the vesicle component and the carrier matrix must meet translational requirements simultaneously.

Clinical-trial landscape: A growing number of ongoing and completed studies are evaluating exosome- or extracellular-vesicle-based products, with mesenchymal stromal/stem cell (MSC)-derived exosomes representing one of the most frequently explored therapeutic platforms in early clinical investigation. However, the current clinical landscape remains highly heterogeneous. Systematic analyses indicate that EV-related trials are still dominated by diagnostic applications—especially in oncology—whereas therapeutic studies are fewer and often differ substantially in donor-cell source, isolation method, characterization workflow, dosing strategy, route of administration, and clinical endpoints. This heterogeneity limits cross-trial comparability and slows evidence-based optimization for regenerative medicine applications [68,69,76,81].

Regulatory classification, manufacturing consistency, and quality control: From a regulatory perspective, therapeutic exosomes are increasingly discussed within a framework similar to biologic medicinal products. Exosome composition is strongly influenced by donor cell source, culture condition, preconditioning protocol, and isolation method, which complicates batch-to-batch consistency and potency assessment. For biomaterial-assisted systems, this problem is further compounded by the need to control material composition, crosslinking density, release behavior, and sterilization compatibility in parallel. Nevertheless, their multicomponent composition, source dependence, and mechanism complexity make conventional chemistry-manufacturing-control assessment more difficult than for many standard biologics. Major unresolved issues include how to define product identity, mechanism-relevant critical quality attributes, potency assays, batch-to-batch comparability, and pharmacokinetics. At a minimum, clinical-grade exosome products should undergo rigorous characterization of donor-cell provenance, culture conditions, isolation and purification procedures, particle concentration and size distribution, canonical EV markers and impurities, cargo composition, sterility, endotoxin, mycoplasma, and storage stability. Consensus frameworks such as MISEV2018 provide an important methodological foundation, but further harmonization is still needed to transform these principles into clinically actionable release criteria and regulatory standards [16,82,83].

Regulatory classification of exosome–biomaterial combination products (FDA/EMA): Because many regenerative exosome platforms integrate a biologically active EV fraction with a hydrogel, scaffold, patch, microneedle, or other delivery matrix, they may not fit cleanly within a single traditional product category. In the United States, such constructs may be regulated as combination products, with primary jurisdiction generally determined by the product’s primary mode of action (PMOA). Thus, an exosome-loaded matrix designed mainly to deliver the biological activity of the EV payload would likely be considered a biologic-led combination product, whereas systems in which the structural or mechanical contribution of the matrix predominates may raise device-led considerations. In the European Union, these systems will more often be evaluated as medicinal products incorporating an integral device constituent, for which Medical Devices Regulation (MDR)-related requirements, including Article 117, may become relevant. By contrast, the “combined ATMP” pathway applies only if a product first fulfills the legal definition of an advanced therapy medicinal product and contains a medical device as an integral part; this classification therefore should not be automatically assumed for most acellular exosome products. From a translational perspective, early clarification of intended use and PMOA, proactive interaction with regulatory agencies, and parallel definition of EV-specific and biomaterial/device-specific quality attributes will be essential.

GMP-compliant production: GMP-compliant manufacturing remains another major bottleneck for translation. Clinical translation requires robust upstream manufacturing, reproducible purification workflows, validated release specifications, and acceptable long-term storage strategies such as optimized cryopreservation or lyophilization. Scalable production requires robust upstream culture systems, ideally serum-free or xeno-free, well-defined master cell banks, reproducible large-scale expansion, closed or semi-closed downstream purification workflows, validated fill-finish procedures, and storage strategies that preserve vesicle integrity and bioactivity. For exosome–biomaterial combination systems, these challenges are compounded by the need to ensure compatibility, sterility, and stability of both components after formulation. Additional barriers include low production yield, process-dependent heterogeneity, co-isolated contaminants, the lack of universally accepted dosing metrics, and incomplete understanding of how freezing, lyophilization, sterilization, or scale-up affect therapeutic potency. Addressing these issues will be essential before exosome-based regenerative therapeutics can move from promising laboratory constructs to reproducible and regulator-ready clinical products [16,76,82].

Source-Dependent Biosafety of Exosomes: Beyond the safety of biomaterial components, the biological safety of exosomes themselves requires careful attention. Exosomes are not compositionally neutral carriers; rather, they may inherit membrane proteins, lipids, and signaling molecules from their parental cells, and their cargo composition may vary with cell source, activation state, culture condition, and engineering method [13]. This creates a source-dependent risk of unintended biological effects, including excessive immunosuppression [14,69], aberrant angiogenic signaling, or the transfer of pro-tumorigenic cues [84,85]. Such concerns are particularly relevant when exosomes are derived from poorly defined, diseased, or tumor-adjacent cells [15]. Therefore, future translational studies should incorporate stringent donor-cell selection, molecular profiling of exosomal surface markers and cargo, batch-to-batch comparability testing, and long-term in vivo biodistribution and toxicology assessment. In addition, safety evaluation should extend beyond acute cytotoxicity to include immune perturbation, fibrosis, and tumor-promoting potential in susceptible models [13,82].

### 4.3. Future Perspectives: From “Pre-Programmed” to “Intelligent Interaction”

Looking ahead, the development of exosome–biomaterial systems will advance towards greater intelligence, precision, and efficiency.

Multi-mechanism Synergy Systems: Future designs will move from delivering single-function exosomes to integrated multifunctional systems capable of simultaneously or sequentially delivering multiple types of exosomes, or exosomes in synergy with drugs, to address the multi-pathway, multi-target complexity of tissue repair [34,38].

“Closed-Loop” Smart Feedback Systems: Most current smart responsive materials are “open-loop.” The ultimate goal is to build “closed-loop” smart feedback systems that can continuously “sense” the dynamic changes in the reparative microenvironment and adaptively adjust the type and dosage of released exosomes, achieving truly personalized therapy [53].

AI-Assisted Rational Design and Personalization: AI-Assisted Rational Design and Personalization: Beyond conventional empirical optimization, AI may become an important bridge between exosome heterogeneity and precision therapy. By integrating EV multi-omics data, physicochemical characterization, surface-marker profiles, biodistribution information, and functional outcomes, machine-learning models may help identify donor-cell subpopulations with superior regenerative potential, prioritize therapeutic cargo molecules and targeting ligands, and optimize biomaterial formulations for retention and stimuli-responsive release. In regenerative medicine, such computational frameworks could be further coupled with patient-specific data—including lesion type, inflammatory status, vascularization, and spatial transcriptomic features—to stratify patients and guide the selection of exosome source, engineering strategy, dose, and treatment schedule. Beyond design, AI may also support manufacturing by improving image-based EV classification, batch consistency assessment, and prediction of critical quality attributes. However, these opportunities are still constrained by small and heterogeneous datasets, inconsistent EV isolation and annotation workflows, limited model interpretability, and the lack of prospective clinical validation. Therefore, future progress will require the parallel maturation of standardized EV datasets, robust computational pipelines, and clinically relevant validation frameworks [62,86,87].

**Figure 4:**
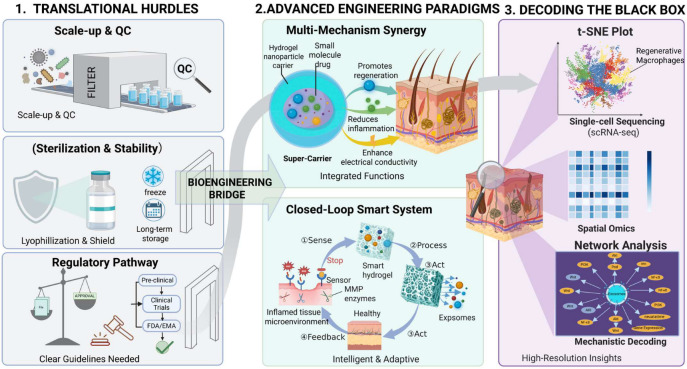
Future roadmap for advancing engineering-allowed exosome therapies: From translational hurdles to mechanistic decoding. The diagram illustrates the “Bioengineering Bridge” connecting current challenges to future clinical success, categorized into three strategic pillars: (1) Translational Hurdles (Left panel): Highlights important barriers to clinical adoption, including Scale-up & Quality Control (QC) for ensuring purity and consistency, Sterilization & Stability strategies (e.g., Lyophilization) for long-term storage, and the establishment of clear Regulatory Pathways (FDA/EMA guidelines) for clinical trials. (2) Advanced Engineering Paradigms (Middle panel): Depicts next-generation design strategies. “Multi-Mechanism Synergy” integrates hydrogels with nanoparticles and small molecules into “Super-Carriers” to simultaneously reduce inflammation and promote regeneration. “Closed-Loop Smart Systems” represent intelligent, adaptive materials that Sense pathological cues (e.g., MMP enzymes), Process the signal, and Act by releasing exosomes on-demand, creating a therapeutic feedback loop. (3) Decoding the Black Box (Right panel): Emphasizes the use of high-resolution omics technologies to describe therapeutic mechanisms. Approaches include Single-cell Sequencing (scRNA-seq) to identify specific regenerative cell subsets (e.g., macrophages), Spatial Omics to map gene expression within the tissue architecture, and Network Analysis to mechanistically decode the molecular signaling pathways activated by exosomes.

New Tools for Mechanistic Insights: Future progress depends on exploring the “black box.” The application of analytical techniques like single-cell RNA sequencing (scRNA-seq) and spatial transcriptomics will allow us to dissect the material–exosome–cell interaction network at an unprecedented resolution. This will help us discover new responsive cell subpopulations [62] and major regulatory pathways [57], thereby providing the scientific rationale for more advanced biomaterial designs. This may improve exosome therapy from a dazzling laboratory gem into an accessible treasure on the clinical shelf (Figure 4).

## Figures and Tables

**Table 1:** Representative biomaterial-assisted exosome delivery systems for regenerative medicine.

Application	Biomaterial System	Exosome Source	Core Strategy	Release/Retention Feature	Model	Main Outcome/Mechanism	Key Limitation/Ref.
Skin wound healing	pH-responsive self-healing antibacterial hydrogel	MSC-derived exosomes	Microenvironment-responsive on-demand release	pH-triggered degradation with sustained local exposure	Diabetic/chronic wound model	Accelerated wound closure, angiogenesis, and re-epithelialization; anti-inflammatory and pro-angiogenic signaling	Limited cross-study comparability of release kinetics; hybrid storage/sterility need optimization [28]
Skin wound healing	ROS-adaptive multifunctional hydrogel	Stem cell-derived exosomes	ROS-responsive self-adaptive release	Oxidative stress-triggered release with reduced premature loss	MRSA-infected diabetic wound model	Improved infected wound repair, inflammation control, and regeneration	Multi-component system increases manufacturing and regulatory complexity [53]
Skin wound healing	Hydrogel loaded with MnO_2_ nanosheets, exosomes, and FGF2	M2 macrophage-derived exosomes	Combined microenvironment modulation and sustained delivery	Coordinated local release of bioactive factors	Diabetic wound model	Enhanced vascularization and granulation; modulation of oxidative stress, inflammation, and angiogenesis	Complex formulation may increase batch variability and QC burden [60]
Skin wound healing	Exosome-embedded hydrogel	Hypoxia-pretreated ADSC-derived exosomes	Sustained release plus donor-cell preconditioning	Prolonged local retention during healing	Diabetic wound model	Enhanced angiogenesis and wound healing; improved pro-regenerative cargo activity	Preconditioning may increase exosome heterogeneity and potency variation [10]
Bone/cartilage regeneration	3D-printed bilayer gradient dECM hydrogel scaffold	Therapeutic exosomes	Structural guidance and zonal regeneration	Sustained release from layered porous construct	Rat osteochondral defect model	Improved cartilage and subchondral bone repair; lineage-specific regeneration	Mechanical validation and scalable fabrication remain challenging [30]
Bone/cartilage regeneration	Injectable mussel-inspired adhesive hydrogel	Exosomes for cartilage repair	Tissue anchoring and endogenous cell recruitment	Prolonged lesion retention with reduced washout	Cartilage defect model	Enhanced defect filling and cartilage regeneration	Strong adhesion may reduce diffusion depth; quantitative retention data remain limited [32]
Bone/cartilage regeneration	3D-printed trabecular titanium scaffold + hydrogel	Hypoxia-induced exosomes	Mechanical support plus sustained release	Long-term local presentation in scaffold–hydrogel composite	Bone defect model	Enhanced osteogenesis and angiogenesis; activation of MAPK/mTOR/HIF-1/VEGF-related pathways	Composite metal–hydrogel systems may face more complex translation and sterilization issues [46]
Bone/cartilage regeneration	High-performance hydrogel with engineered exosomes	Engineered exosomes	Source engineering plus local retention	Sustained local delivery with improved bioactivity	Diabetic bone regeneration model	Boosted bone regeneration and ER homeostasis	Engineered exosomes require stricter cargo characterization and reproducibility control [40]
Bone/cartilage/OA	Polypeptide hydrogel microcarriers	Therapeutic exosomes	Exosome delivery plus endogenous stem cell homing	Localized retention with dual-function microcarriers	Osteoarthritis model	Improved cartilage repair and joint microenvironment modulation	Added functionalization increases formulation complexity [48]
Myocardial repair	Aptamer-functionalized DNA hydrogel	MSC-derived exosomes	Specific capture and local sustained delivery	Aptamer-assisted retention and non-destructive loading	Myocardial infarction model	Improved cardiac function, reduced apoptosis/fibrosis; regulation of Bcl-2/Bax and TGF-β/Smad-related signaling	DNA hydrogel cost, stability, and scale-up need further study [39]
Myocardial repair	Conductive polypyrrole/chitosan hydrogel	Human endometrial MSC-derived exosomes	Injectable conductive hydrogel with sustained release	Sustained release in dynamic myocardium	Myocardial infarction model	Enhanced angiogenesis, anti-apoptosis, and cardiac repair; activation of EGF/PI3K/AKT signaling	Multifunctional conductive systems increase CMC and regulatory burden [61]
Nerve regeneration	Electroconductive hydrogel	BMSC-derived exosomes	Conductive scaffold plus sustained neurotrophic delivery	Sustained release with structural/electrical guidance	Spinal cord injury model	Improved axonal growth, remyelination, and functional recovery; NF-κB and PTEN/PI3K/AKT/mTOR-related regulation	Translation requires better standardization of conductivity, architecture, and EV potency [31]
Nerve regeneration	ROS-responsive EV-integrated hydrogel	Engineered extracellular vesicles	Intelligent ROS-triggered release	Injury-site oxidative stress accelerates EV release	Spinal cord injury model	Enhanced neuroprotection, angiogenesis, and anti-inflammatory repair	Responsive chemistry and long-term degradation safety require validation [29]
Nerve regeneration	Hydrogel-assisted local exosome delivery	CD271^+^CD56^+^ BMSC-derived exosomes	Precision donor-cell selection plus local delivery	Sustained local presentation with optimized cargo	Spinal cord injury model	Enhanced axonal regeneration via the miR-431-3p/RGMA axis	Precision source selection increases characterization burden and cost [62]

Abbreviations: ADSC, adipose-derived stem cell; BMSC, bone marrow mesenchymal stem cell; dECM, decellularized extracellular matrix; EV, extracellular vesicle; MSC, mesenchymal stem cell; OA, osteoarthritis; ROS, reactive oxygen species. Note: Because different studies used different dosing units, release assays, and tracking methods, the release/retention characteristics are summarized here descriptively rather than as directly comparable quantitative values.

## Data Availability

No new data were created or analyzed in this study. Data sharing is not applicable.
